# Thank you to our reviewers

**DOI:** 10.1017/cts.2024.8

**Published:** 2024-02-05

We are indebted to the expert referees who have volunteered their time to review submissions for the Journal of Clinical and Translational Science in 2023. The editors, authors and readers are immensely grateful for their thoughtfulness and expertise that have served our journal well.

Zainab Abedin

Stacey Adam

Joan Adamo

Andrew Admon

Samuel E. Adunyah

Asya Agulnik

Tabia Akintobi

Hisam Alahdab

Allan Albig

Ayham Alkhachroum

Caitlin Allen

Emily Anderson

LaKesha N. Anderson

Nicki Apaydin

Danit Ariel

Jennifer Armstrong

Gladys Asiedu

Irfan Asif

Sanja Avramovic

Justin Baca

Shasha Bai

Chighaf Bakour

Laura Balis

Joyce Balls-Berry

Lauren Balmert

Karen Bandeen

Shweta Bansal

Claudia Baquet

Daniel M. Barnett

Jason Bates

Ana Baumann

John Baumann

Tara Bautista

Michael Becich

Mary Becker

Lauren Bednarz

Joseph Bednash

Jasmine Bell

Gordon R. Bernard

Steven L. Bernstein

Michael Beyerlein

Jiang Bian

Barbara Bierer

Heather Billings

Leslie Boone

John Borghi

Christine Borgman

Barbara Bowers

Barrett Bowling

Kathleen T. Brady

Carolyn Bramante

Allan R. Brasier

Miriam A. Bredella

Ahnalee Brincks

Amanda Marie Brock

Tabetha A. Brockman

Birgitta Brott

Crystal E. Brown

Timothy Buchman

Elizabeth Burner

John B. Buse

Loretta M. Byrne

Christopher Cambron

Patricia Candelaria

Marlene Cano

Nichole Carlson

Savanna Carson

Lori Carter-Edwards

Louis Carvajal

Shannon Casey

David Center

Johanna Chesley

James J. Cimino

Nichelle L. Cobb

Gretchen Colbenson

Barry Coller

John Connett

Sara Conroy

Dan Michael Cooper

Lisa D. Cooper

Linda Cottler

Valerie Cotton

Jessica Cranfill

Timothy N. Crawford

Jennifer Croker

Mollie Cummins

Jennifer Cunningham-Erves

Yifang Dang

Suzanne Danhauer

Proleta Datta

Denise Daudelin

Gaurav Dave

Pamela B. Davis

Wilfredo De Jesus

Amos De Jong

Emilia De Marchis

Guilherme DelFiol

Erica Denhoff

Manisha Desai

Jennifer Devoe

Judith Dexheimer

Earl (Ray) Dorsey

Jamie Mihoko Doyle

Ann M. Dozier

Emily Dressler

Nishita Dsouza

David L. DuBois

Zachary Dyer

Carrie Dykes

Brenda Eakin

Heather Edelblute

Milton Mickey Eder

Peter L. Elkin

Vicki L. Ellingrod

Felicity T. Enders

Kolaleh Eskandanian

Estela Estape

Julio Facelli

Megan L. Falsetta

Hank Farrar

Titilope Fasipe

Layla Fattah

Josh Fessel

Joseph Finkelstein

Daniel Ford

Evan Forman

Deborah M. Fournier

Stephanie Freel

Christopher Frei

Derrick Fung

Janice L. Gabrilove

Byron Gajewski

Eden Gallegos

Mohit Ganguly

Cheryl Gatto

Mugur Geana

Ken Gersing

Jesse Gibson

Cynthia J. Girman

Kristine Glauber

Kelly Gleason

Rachel Gold

Elizabeth H. Golembiewski

Ruth Gotian

Angela Griffiths

Ronnie Guillet

İskender Gün

Christine Gunn

Serena Guo

Yi Guo

Mikel Gurrea-Rubio

Erinn Hade

Nathaniel Hafer

Wyatte C. Hall

Ann Hamilton

Megan E. Hamm

Daniel F. Hanley

Alexandra Hanlon

Courtney Hanny

Md Rejuan Haque

Michael Harnar

Paul A. Harris

Micah Hartwell

Xing He

Anna Heath

Elizabeth Heitman

Shirley L.T. Helm

Erik Henricson

Rachel Hess

Patrice Hicks

Elizabeth S. Higgs

Hwanhee Hong

Christoph Hornik

Lauren Houston

Daniel Hsia

Xuemei Huang

Yu Huang

David H. Ingbar

Christy Irving

Rami Issa

Michael A. Jacobs

Laura P. James

Carey Jansen

Erick Jenkins

Rebecca N. Jerome

Ann Johnson

Carolynn Thomas Jones

Maya Juarez

Felichism W. Kabo

Alexander Mark Kaizer

Heather Cecille Kaplan

Wahida Karmally

Daniel Karp

Al-Faraaz Kassam

Ken Kawamto

Jacob Kean

Heidi Keeler

Thomas H. Kelly

Suzanne Kennedy

Nick Kenyon

Philip Kern

Anthony Keyes

Sawsan Khuri

Amy Kilbourne

Joanne Kim

Yongjoo Kim

Robert Kimberly

Arati Kiran Kanchi

Kirsten Klein

Lisa M. Klesges

Heather Klitzman

Jacqueline Knapke

Boyd Knosp

Christa Koenig

Rhonda G. Kost

Joseph A. Kotarba

Helena Kraemer

Edward Krupat

Jack Kues

Brooks Kuhn

Bethany M. Kwan

Jeffrey Kwong

Kate LaForge

Marcus Lambert

Michelle M. Lamere

Taylor Lane

Marianna LaNoue

Bernard LaSalle

MinJae Lee

Harold Lehmann

Chenyu Li

Junjing Lin

Ruitao Lin

Christopher Lindsell

Craig Lipset

Jialin Liu

Qi Liu

Gaetano Romano Lotrecchiano

Tammy L. Loucks

Emily M. Lund

Kevin Lybarger

Michael Lyons

Camille Anne Martina

Karen G. Martinez

Sandra K. Masur

Colleen A. Mayowski

Donald McClain

Wayne T. McCormack

Brian McCourt

Richard McGee

Scott McGuinness

Linda McManus

Laura McNay

Kathleen McTigue

Emma Anne Meagher

Valentina Medici

Christina Mehta

Tara G. Mehta

Jareen Meinzen-Derr

Joseph Menetski

Robin Mermelstein

Peter Mesenbrink

Oanh Meyer

Lloyd Michener

lucio Miele

Ki Milligan

Jungwon Min

Brooke EE Montgomery

Sean Mooney

Clai Morehead

Elaine H. Morrato

Cynthia Morris

Ahmad Mourad

Jessica Mozersky

Rahma Mungia

Susan Lynn Murphy

Maureen Murtaugh

Donald E. Nease

Rocio Norman

Keith C. Norris

Katia Noyes

Ruth O’Hara

Ivan Oransky

Mila Ortigoza

Robert A. Oster

Debra Oto-Kent

Avik Pal

Vincent J. Palusci

Hannah Park

Michele Patel

Cecilia Patino-Sutton

Rita Patterson

Thomas Pearson

Clara M. Pelfrey

Senaka Peter

Ryan Peterson

Jimmy Phuong

Matthew Pietryka

Harold Pincus

Tiffany Danielle Pineda

Mark Pletcher

Roy Poblete

Gina-Maria Pomann

William Powderly

Janelle Pugashetti

Susan Pusek

Maya Indira Ragavan

Megha Ramaswamy

Martha Ravola

Sarah Read

David Redden

Dominic N. Reeds

Judith Regensteiner

lora Reineck

Ralph Renger

David Resnik

Naomi Riches

Dana Rizk

Paula Roberson

Sarah Robertson

Donita L. Robinson

Jamie Robinson

Ryan Robinson

Mitra Rocca

Frank W. Rockhold

Max Rohde

Betsy Rolland

Gretchen Roman

Morteza Roodgar

Baback Roshanravan

Heather Ross

Reza Rostami

Mary-Tara Roth

Russell Rothman

Jacob Rowan

Doris Rubio

eugene sadhu

Eduardo Salas

Mary Sammel

Elias Samuels

Saul Schaefer

Jonathan Schildcrout

Lisa Schimmenti

Mike Schivo

Joseph Schmelz

Susanne Schmidt

Denise Scholtens

Norah Schwartz

Robert Sege

Harry Selker

Robert Sepanski

Jackilen Shannon

Eugene D. Shapiro

Yashoda Sharma

Fadia Tohme Shaya

Payam Sheikhattari

Gan Shi

Heather Siefkes

Rani Singh

Megan Kasimatis Singleton

Davey Smith

Justin Dean Smith

Melissa Smith

Jessica Snowden

Denise C. Snyder

Anthony Solomonides

Stephen Sonstein

Christine Sorkness

Gerald Moose Stacy

Scott Steele

Mark Stein

John F. Steiner

Julie Stephens

Noreen Stephenson

Andrea Stevens

Kathleen R. Stevens

Thomas G. Stewart

Jackie Stocking

Kayla Stover

A. H. Strelnick

Laura Jane Sugarwala

David Sullivan

Jan Sullivan

Katherine Sward

Umberto Tachinardi

Meagan Talbott

Melanie Taverna

Sandra Taylor

Diana Thomas

Adrian Thorogood

Jennifer Tiffany

Beth B. Tigges

Martha Tillson

Fode Tounkara

Mark Trusheim

Laurene Tumiel Berhalter

Emma Tumilty

Louise Underdahl

PJ Utz

Sangeetha Vadakke-Madathil

John VanBuren

Rebecca Vanderpool

Eleni Okeanis Vaou

Aditi Vasan

Elizabeth Vasile

Alice Villalobos

Tuhin Virmani

Alfred Vitale

Boris Volkov

Sarah Vordenbergy

Russ Waitman

Daniel Walker

Jeffery Walker

Nina Wallerstein

Guanbo Wang

Yanshan Wang

Maranda Ward

Michael J. Ward

Gloria Warner

Molly Wasko

Anne Marie Weber-Main

Lai Wei

Kate Welshons

Shaina Willen

Karen Wilson

Joyce Winkler

Eli Wisdom

Nellie Wixom

Michel Woodbury

Kevin Wooten

Yonghui Wu

Jiawei Xu

Donglin Yan

Fei Yu

Maham Zahid

Lu Zhang

Runzhi Zhang

Yanan Zhang

Brenda Zierler

Andrew Zinn

Deborah Zucker

Diana M. Zuckerman

